# From Virtual Modelling to Smarter Endodontic Innovation: In Silico Research for Translational Decision Making

**DOI:** 10.1111/iej.70233

**Published:** 2026-07-22

**Authors:** José Evando da Silva‐Filho, Eduardo Diogo Gurgel‐Filho

**Affiliations:** ^1^ Department of Dental Radiology and Imaging University of Fortaleza Fortaleza Ceará Brazil; ^2^ Department of Endodontics University of Fortaleza Fortaleza Ceará Brazil

**Keywords:** biomaterials, computational modelling, endodontics, in silico research, pathological pathways, simulation

## Abstract

**Aim:**

To discuss how in silico research may function as an early translational decision layer in endodontics by supporting the prioritisation of hypotheses, technologies and experimental conditions before biological and clinical validation.

**Summary:**

Endodontic outcomes emerge from interacting anatomical, mechanical, chemical, microbial and host‐related variables. Conventional studies using extracted teeth, artificial canals, selected microbial models and standardised tests remain essential, yet cannot represent the full variability encountered in clinical settings. In silico approaches, including finite element analysis, computational fluid dynamics, probabilistic simulations, molecular modelling and predictive toxicology, may extend preclinical investigation by mapping plausible scenarios across pathological pathways, instrument performance, biomaterials and irrigation. Their contribution depends on a clearly defined context of use, justified input data, transparent assumptions, verification, validation, sensitivity analysis and uncertainty assessment. Computational outputs should be interpreted according to the question the model was designed to address and should guide subsequent experimental selection rather than be treated as independent evidence of clinical effectiveness.

## 1 Introduction

Endodontics is a core field of dentistry dedicated to the prevention, diagnosis and treatment of diseases affecting the dental pulp and periapical tissues. Its main clinical purpose is to preserve teeth by controlling infection, reducing inflamed or necrotic tissue, disinfecting complex root canal systems and creating conditions for periapical healing (Nair 2004, 2006; Siqueira Jr and Rôças 2008; Ricucci et al. 2010). Although these goals may appear straightforward, endodontic treatment depends on a demanding sequence of biological, chemical and mechanical procedures.

Files must shape irregular canals without fracture or excessive dentine removal; irrigants must reach inaccessible anatomical regions without unacceptable toxicity; biomaterials must seal, interact with dentine and remain stable in a moist biological environment; and therapeutic strategies must deal with microbial persistence, host response and inflammatory pathways (Nair 2004, 2006; Siqueira Jr and Rôças 2008; Ricucci et al. 2010; de Sousa‐Neto et al. 2018; Chien et al. 2021; Barros et al. 2014; Boutsioukis, Gogos, et al. 2010). For these reasons, experimentation remains indispensable. These interventions must be tested, understood and validated before they influence clinical decisions.

Computational approaches are increasingly influencing biomedical research. Computational modelling, molecular docking, network pharmacology, finite element analysis, computational fluid dynamics and probabilistic simulations are changing how hypotheses are generated, how devices are designed and how biological systems are interpreted (Pappalardo et al. 2019; Morrison et al. 2018; Viceconti et al. 2021; Pathmanathan et al. 2024). Endodontics is particularly suited for this shift because its main challenges involve anatomical variability, instrument mechanics, material‐tissue interactions, irrigant dynamics, microbial ecology and pathological pathways (Nair 2004, 2006; Siqueira Jr and Rôças 2008; Ricucci et al. 2010; de Sousa‐Neto et al. 2018; Chien et al. 2021; Barros et al. 2014; Boutsioukis, Gogos, et al. 2010; Pappalardo et al. 2019; Morrison et al. 2018; Viceconti et al. 2021; Pathmanathan et al. 2024).

Thus, this clinical commentary aims to discuss how in silico research should be incorporated as an early translational decision layer in the field, allowing technologies and therapeutic strategies to be virtually modelled, critically prioritised and selectively validated before clinical translation. The purpose is not to propose simulation as a replacement for conventional research, but to clarify how it may improve experimental design across different endodontic domains.

## 2 Report

### 2.1 Why Experimental Endodontics Needs a Broader Decision Layer?

Preclinical investigations frequently depend on extracted teeth, artificial canals, single‐species biofilms or standardised mechanical and chemical conditions (Siqueira Jr and Rôças 2008; Ricucci et al. 2010; de Sousa‐Neto et al. 2018; Chien et al. 2021; Barros et al. 2014; Boutsioukis, Gogos, et al. 2010). These models provide control, reproducibility and evidence that cannot be ignored, but they represent only a restricted portion of the clinical landscape. Testing 50, 100 or even 200 teeth can answer specific questions, but such numbers cannot reproduce the full variability that a new concept may face in clinical use. In silico research may help address this gap by moving endodontic research from isolated testing to broader scenario mapping (Pappalardo et al. 2019; Morrison et al. 2018; Viceconti et al. 2021; Pathmanathan et al. 2024). The main question is therefore not whether virtual conditions can replace physical specimens, but whether modelling can help identify which specimens, conditions and experimental designs are most informative.

Nonetheless, simulating a broad range of virtual conditions should not be interpreted as a simple computational shortcut. Constructing a credible model that approximates a real clinical or laboratory scenario requires careful definition of geometry, material properties, boundary conditions, biological assumptions, numerical settings and outcome measures. The computational burden may increase substantially when additional anatomical variation, higher resolution, coupled mechanical and biological processes or time‐dependent phenomena are introduced. Furthermore, computational models should not replace biological validation. Simulated performance should not be treated as clinical effectiveness. Their role is more specific: to help researchers understand mechanical behaviour, material performance, chemical delivery, microbial interactions and pathological mechanisms before experiments move forward without a clear mechanistic direction (Pappalardo et al. 2019; Morrison et al. 2018; Viceconti et al. 2021; Pathmanathan et al. 2024). In this sense, in silico research may operate as a translational decision layer, connecting hypothesis generation, virtual testing, biological validation and clinical translation. This broader logic is summarised in Figure 1. The main conceptual approaches encompassed by in silico research, together with their core principles, data requirements and typical outputs, are summarised in Table 1.

**FIGURE 1:**
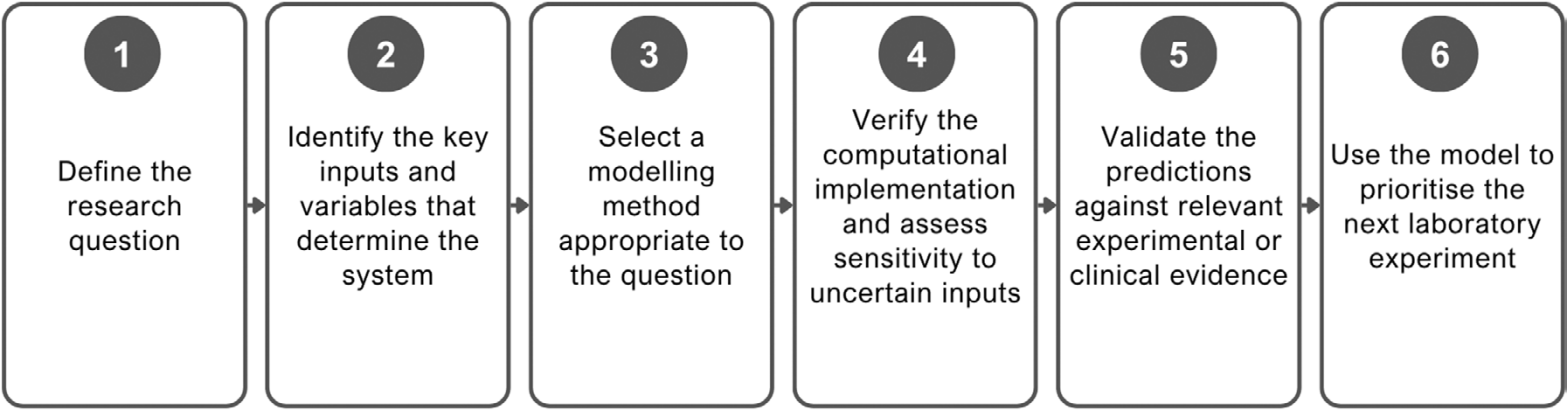
Workflow for in silico research as a translational decision layer in endodontics. Legend for Figure 1: The workflow begins by defining a research question and identifying the input variables that determine the system under investigation. An appropriate modelling method is then selected according to the intended context of use. The computational implementation should be verified and the influence of uncertain inputs should be assessed before predictions are validated against relevant experimental or clinical evidence. The resulting model may then be used to prioritise the next laboratory experiment, allowing physical validation to focus on the questions and conditions most likely to be informative.

**TABLE 1:** Core conceptual approaches within in silico endodontic research.

Approach	Core principle	Main data requirements	Typical outputs
Finite element analysis	Numerical representation of physical responses within complex geometries	Geometry, material properties, loading conditions, contact assumptions, and boundary conditions	Stress, strain, displacement, deformation, and contact pressure
Computational fluid dynamics	Numerical representation of fluid motion and transport	Canal geometry, fluid properties, flow conditions, needle position, and boundary conditions	Velocity, pressure, wall shear stress, and flow distribution
Probabilistic modelling	Representation of uncertain parameters as distributions	Input distributions, correlations, assumptions, and scenario definitions	Uncertainty intervals, sensitivity profiles, and outcome distributions
Molecular docking and molecular dynamics	Structure‐based estimation of molecular interactions and stability	Molecular structures, binding sites, interaction settings, and solvent assumptions	Binding poses, interaction scores, and molecular stability
Systems biology and network pharmacology	Integration of biological datasets and interaction networks	Transcriptomic, proteomic, microbial, and pathway data	Network associations, enriched pathways, and candidate targets
Predictive toxicology	Computational estimation of potential safety signals	Chemical descriptors, molecular properties, and exposure assumptions	Toxicity alerts, hazard estimates, and candidate ranking
Image‐based anatomical modelling and virtual cohorts	Digital reconstruction and generation of anatomical scenarios	Imaging data, anatomical measurements, and population distributions	Three‐dimensional geometries, anatomical descriptors, and virtual scenario sets

### 2.2 Statistical Power Is Not Clinical Representativeness

Sample size calculation illustrates this distinction. In a conventional endodontic study, researchers may use an a priori calculation to estimate the minimum number of specimens required to detect a predefined difference between groups. Tools such as G*Power (Heinrich‐Heine‐Universität, Düsseldorf, Germany) support this process by calculating sample size and power for several statistical tests under assumptions such as effect size, alpha level, statistical power and allocation ratio (Faul et al. 2007; Kang 2021). Such calculations are appropriate for hypothesis testing because they indicate whether a study is likely to detect an expected effect under controlled conditions. However, statistical power does not ensure representativeness, because a sample may be statistically adequate and still be anatomically, biologically, chemically or mechanically narrow. For example, 20, 40 or 100 specimens may be sufficient to detect a difference in cyclic fatigue, irrigant penetration, antimicrobial activity, bond strength or material behaviour. The same sample cannot represent the full range of canal curvatures, apical diameters, dentine thicknesses, biofilm architectures, inflammatory profiles, operator conditions and clinical handling scenarios (Siqueira Jr and Rôças 2008; Ricucci et al. 2010; de Sousa‐Neto et al. 2018; Chien et al. 2021; Barros et al. 2014; Boutsioukis, Gogos, et al. 2010). Power detects effects, whereas modelling explores variability. Computational modelling can therefore complement this calculation by expanding the scenario space before physical validation.

Parameters such as canal curvature, canal diameter, dentine thickness, file geometry, irrigant flow, material viscosity, diffusion behaviour, microbial load and host‐related variables can be modelled as distributions rather than fixed values (Pappalardo et al. 2019; Morrison et al. 2018; Viceconti et al. 2021; Pathmanathan et al. 2024; Velikova et al. 2024). Monte Carlo simulations and related probabilistic approaches may then generate multiple virtual scenarios, helping preserve time, resources and investment for questions with biological and clinical relevance (Velikova et al. 2024). Nevertheless, a million virtual scenarios do not automatically represent a million clinically plausible teeth. The relevance of a virtual cohort depends on the quality and diversity of the data used to generate it. Micro‐computed tomography datasets may support the creation of anatomically informed virtual canals, while probabilistic approaches may vary canal dimensions, curvatures, dentine thicknesses and other measurable parameters (de Sousa‐Neto et al. 2018; Velikova et al. 2024).

### 2.3 Model Credibility Determines Whether Simulation Produces Useful Knowledge

The practical value of in silico research depends on model credibility. A model should first have a clearly stated context of use and researchers must define its intended purpose before interpreting its outputs. Claims derived from the model should remain consistent with this purpose. A model developed to compare stress concentration between two instruments cannot independently establish clinical fracture incidence, just as a model developed to estimate irrigant flow cannot independently demonstrate microbial elimination. Verification and validation should be considered separately: verification confirms that the computational implementation solves the intended mathematical model correctly, whereas validation establishes whether its predictions are consistent with relevant experimental or clinical evidence (Pappalardo et al. 2019; Morrison et al. 2018; Viceconti et al. 2021; Pathmanathan et al. 2024).

A credible model should also describe the origin and uncertainty of its input parameters. Geometry, material properties, loading conditions, fluid behaviour, chemical concentrations, microbial conditions and biological assumptions should be justified rather than selected solely for convenience. Sensitivity analysis should identify which variables exert the greatest influence on the outputs, whereas uncertainty quantification should determine whether the conclusions remain stable when plausible input variation is introduced. Calibration and validation data should be distinguished whenever possible and sufficient methodological detail should be reported to permit independent reproduction. These requirements do not make simulation less attractive; rather, they make its application safer and more informative.

### 2.4 In Silico Research as a Translational Decision Layer

In silico research should not be viewed only as a way to increase sample size or accelerate preclinical testing. Its value lies in creating a decision layer between biological complexity, technological development and experimental validation (Pappalardo et al. 2019; Morrison et al. 2018; Viceconti et al. 2021; Pathmanathan et al. 2024). This may help define which hypotheses deserve attention, which variables should be prioritised, which failure modes should be anticipated and which experimental models are most appropriate for the next step. Endodontic innovation follows different routes. Some questions begin with disease mechanisms (Nair 2004, 2006; Siqueira Jr and Rôças 2008; Ricucci et al. 2010), others begin with technology (de Sousa‐Neto et al. 2018; Chien et al. 2021; Barros et al. 2014; Boutsioukis, Gogos, et al. 2010; Pappalardo et al. 2019; Morrison et al. 2018; Viceconti et al. 2021; Pathmanathan et al. 2024).

In both routes, computational modelling can organise complexity before resources are invested in physical, biological or clinical testing. In silico research may therefore function as a filter, a map and a stress test at the same time. It filters weak hypotheses, maps anatomical and biological variability and exposes promising strategies to clinically relevant or extreme conditions before bench validation (Pappalardo et al. 2019; Morrison et al. 2018; Viceconti et al. 2021; Pathmanathan et al. 2024). Instead of asking only whether a technology works under one controlled condition, researchers may first ask where it works, where it fails, why it fails and which conditions really deserve further investigation (Figure 2). The potential contribution of in silico research across the main endodontic domains, together with its interpretative limitations and subsequent validation needs, is summarised in Table 2.

**FIGURE 2:**
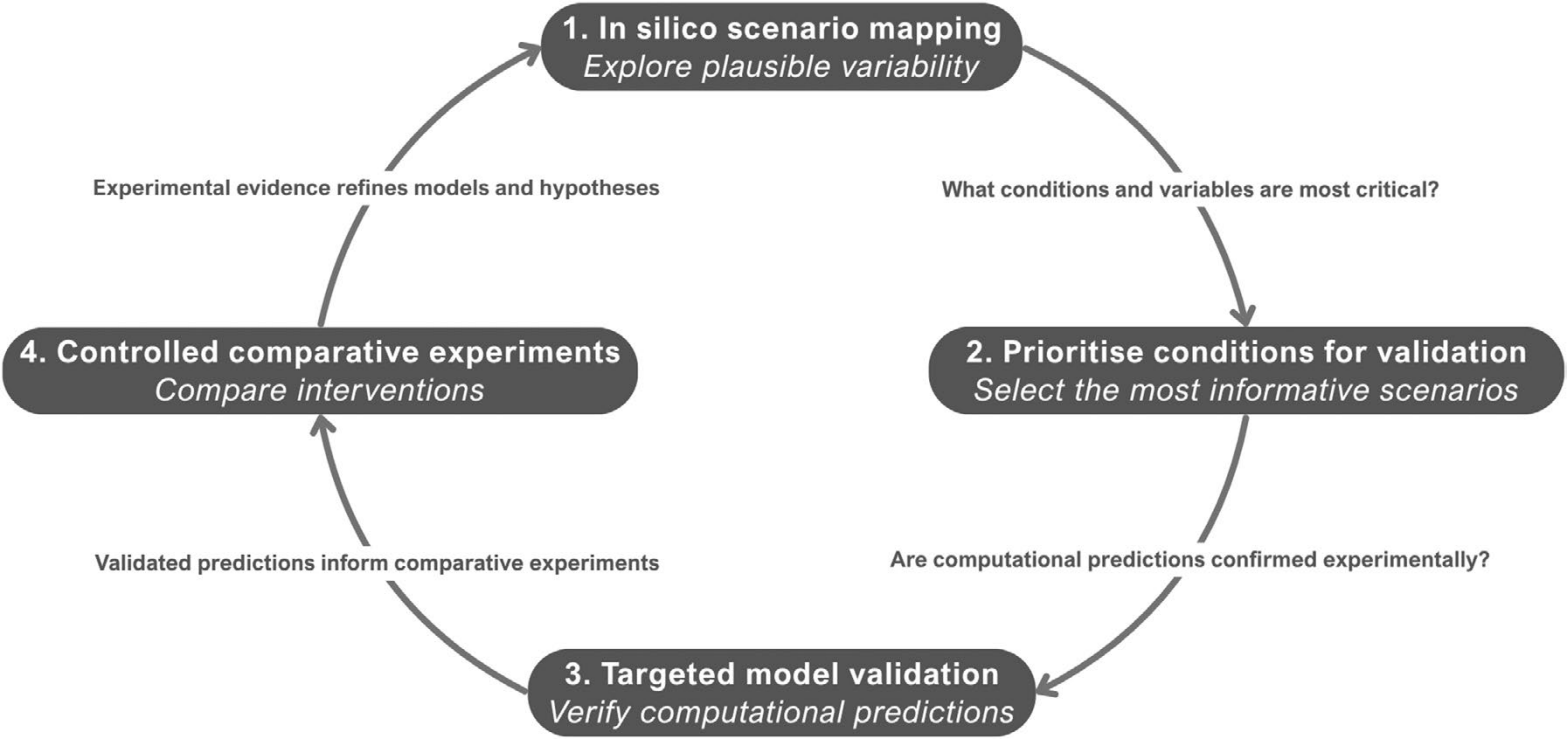
Cyclical integration of in silico scenario mapping and experimental validation in endodontic research. Legend for Figure 2: In silico scenario mapping explores plausible anatomical, mechanical, chemical, microbial and host‐related variability to identify the conditions and variables most likely to influence predicted outcomes. These outputs are used to prioritise the most informative scenarios for validation. Targeted model validation then verifies whether computational predictions remain valid under relevant physical and biological conditions. Once these predictions are confirmed, controlled comparative experiments provide standardised comparisons of materials, techniques or interventions. The resulting experimental evidence informs and refines models and hypotheses, initiating a new cycle of more focused translational research.

**TABLE 2:** Potential contributions, interpretative limitations and subsequent validation needs of in silico research across endodontic domains.

Endodontic domain	Potential role	Relevant outputs	Interpretative limitations	Required subsequent validation
Persistent infection and inflammatory pathways	Which targets or molecules deserve further investigation	Candidate targets, pathway associations and ranked compounds	Therapeutic benefit or control of apical disease	Biochemical, microbial, cellular and tissue‐based studies
Instrument design and mechanical risk	Which designs or anatomies require physical testing	Critical stress regions, deformation patterns and high risk scenarios	Clinical fracture incidence or universal clinical performance	Cyclic fatigue, torsional resistance and shaping studies
Biomaterial development	Which components or formulations should move forward	Candidate ranking, predicted interactions, safety alerts and diffusion patterns	Clinical sealing, biocompatibility, antimicrobial action or durability	Physicochemical, microbiological and biological testing
Irrigation and chemical delivery	Which protocols and anatomical conditions deserve ex vivo investigation	Limited delivery zones, pressure patterns and comparative flow behaviour	Biofilm removal, tissue dissolution or clinical safety	Flow, pressure, chemical, biofilm and ex vivo studies
Anatomical variation and virtual cohorts	Which anatomical conditions may expose failure or uncertainty	Scenario distributions, sensitivity profiles and failure zones	Full clinical representativeness	Comparison with independent anatomical datasets and extracted teeth studies

## 3 Discussion

### 3.1 Pathological Pathways and Therapeutic Targets

Endodontic disease is not only a mechanical or chemical problem. It is also driven by microbial persistence, host response, inflammatory mediators, bone resorption, immune regulation and tissue repair. Apical periodontitis, persistent infection, post‐treatment disease and regenerative failure involve pathways connecting microorganisms, host cells, cytokines, osteoclastogenesis, extracellular matrix remodelling and healing dynamics (Nair 2004, 2006; Siqueira Jr and Rôças 2008; Ricucci et al. 2010; Milojevic et al. 2024). These interactions create a strong opportunity for in silico research, since systems biology, network pharmacology, molecular docking, molecular dynamics, transcriptomic analysis, proteomic integration and pathway modelling may help identify relevant therapeutic targets (Pappalardo et al. 2019; Meng et al. 2011). Computational methods may prioritise microbial enzymes or receptors as antimicrobial targets, identify host pathways associated with persistent periapical inflammation or screen molecules with potential to modulate infection and repair (Meng et al. 2011). They may also integrate datasets from periapical lesions, pulp inflammation, regenerative procedures and microbial communities. Still, caution remains necessary. A predicted pathway is not automatically a therapeutic solution and favourable docking does not make a molecule an effective intracanal medication. These methods are useful because they reduce uncertainty and guide biological validation toward more plausible targets.

However, the biological complexity of these processes remains a major limitation. Although computational models can incorporate selected host, microbial and environmental variables, they cannot fully reproduce the dynamic interactions that determine apical disease or healing across different clinical scenarios. Their most defensible contribution is therefore to prioritise hypotheses for further investigation rather than to provide complete models of disease progression or treatment response. In therapeutic target modelling, the intended output should be clearly defined, whether it is a candidate target, a predicted molecular interaction, a network association or a possible host‐modulating strategy. Any subsequent translational claim should depend on progressive validation through biochemical, microbial, cellular and tissue‐based evidence.

### 3.2 Files, Anatomy and Mechanical Performance

Endodontic instruments are strong candidates for in silico research because a file is not merely a metal object with a specific design. It is a moving device operating inside a narrow, curved and anatomically variable space while being exposed to torsion, cyclic fatigue, bending, friction and repeated contact with canal walls. Its performance depends on alloy composition, heat treatment, taper, cross‐sectional geometry, pitch, tip design, surface treatment, kinematics, canal curvature, canal diameter, operator use and the number of cycles imposed during instrumentation. Conventional tests remain indispensable, but each captures only part of the clinical problem: a cyclic fatigue test in one artificial canal does not represent all curvatures and radii, a torsional resistance test does not represent all anatomical constraints and a shaping study in a limited number of teeth cannot predict performance across a broader spectrum of canal morphologies (de Sousa‐Neto et al. 2018; Chien et al. 2021; Scattina et al. 2015; Abdelhafeez 2024).

Finite element analysis and related simulations may therefore broaden the developmental phase by estimating stress distribution, deformation patterns, torsional risk, fatigue‐prone regions and design weaknesses under multiple virtual conditions. Digital prototypes may be tested across different curvatures, radii, apical diameters, canal shapes, dentine thicknesses and access cavity designs before physical manufacturing (Chien et al. 2021; Scattina et al. 2015; Abdelhafeez 2024). The result is not final proof of performance, but a better experimental question, as stress concentration, deformation and predicted fatigue behaviour remain dependent on assumptions regarding material properties, contact conditions, canal geometry and kinematics. Virtual simulation may indicate which design is unlikely to perform well, which anatomy represents higher fracture risk and which mechanical condition deserves physical validation, but it cannot independently establish how an instrument will behave during clinical use.

### 3.3 Biomaterials and Material‐Tissue Interactions

Endodontic biomaterials should be understood as clinically active systems rather than isolated chemical products. Their performance emerges from the interaction between the formulation and the root canal environment, including dentine, moisture, tissue remnants, biofilms, host tissues and clinical handling. Clinical suitability cannot therefore be inferred from an isolated property. A material may show favourable flow, bioactivity or antimicrobial potential, yet remain unsuitable when its sealing ability, stability, biological response or degradation behaviour is compromised. These properties should be interpreted as interconnected elements of the same clinical problem (Barros et al. 2014; Capuano et al. 2023). In silico methods may help explore these connections before extensive physical testing by estimating interactions between antimicrobial compounds and microbial targets, indicating early safety concerns through predictive toxicology, modelling molecular movement through dentine or biofilms and simulating viscosity, porosity, setting reactions, degradation patterns and ion release under different conditions (Pappalardo et al. 2019; Meng et al. 2011).

This approach is useful for sealers, hydraulic cements, regenerative scaffolds, nanoparticles, drug‐loaded systems, antimicrobial peptides, natural compounds, repurposed drugs and intracanal medications (Barros et al. 2014; Capuano et al. 2023). Instead of testing many formulations empirically, researchers may first identify which candidates are mechanistically plausible and which present obvious limitations. A simulated biomaterial should not be considered clinically effective, since the behaviour of a biomaterial cannot be inferred from one isolated component or predicted interaction. A compound that appears favourable in a docking or toxicity model may behave differently after incorporation into a complete formulation and exposure to dentine, moisture, tissue fluid, microbial challenge and clinical handling. Simulation may make biomaterial development less dependent on blind experimentation and more dependent on mechanistic understanding, while still requiring physicochemical, microbiological and biological validation before any claim regarding clinical performance can be made.

### 3.4 Irrigation, Biofilm Behaviour and Chemical Delivery

Endodontic disinfection depends on chemistry and delivery. An irrigant must have antimicrobial or tissue‐dissolving potential, but it must also reach critical regions of the canal system at an effective concentration and for a sufficient period. Fins, isthmuses, lateral canals, apical ramifications, oval extensions and dentinal tubules are not equally accessible to irrigating solutions (Ricucci et al. 2010; Boutsioukis, Gogos, et al. 2010; Shen et al. 2023; Boutsioukis, Verhaagen, et al. 2010). Computational fluid dynamics may simulate irrigant flow, apical pressure, wall shear stress, vapour lock, needle insertion depth, activation protocols, canal enlargement and anatomical complexity, indicating areas more likely to remain undertreated and strategies more likely to improve irrigant distribution (Boutsioukis, Gogos, et al. 2010; Shen et al. 2023; Boutsioukis, Verhaagen, et al. 2010).

Biofilm simulation is also relevant because endodontic biofilms are not homogeneous targets. Different species may occupy different ecological niches and their response to irrigants or intracanal medications may depend on matrix protection, local concentration, oxygen tension, nutrient availability, metabolic state and interspecies interactions (Nair 2006; Siqueira Jr and Rôças 2008; Ricucci et al. 2010). A single‐species planktonic model may be useful for screening, but it cannot represent the ecological structure of an infected root canal (Nair 2006; Siqueira Jr and Rôças 2008; Ricucci et al. 2010). In silico approaches can help decide which anatomy is most difficult to disinfect, which irrigation protocol deserves ex vivo validation, which concentration range is plausible before cytotoxicity becomes unacceptable and which combination should not be prioritised because the predicted benefit is weak (Boutsioukis, Gogos, et al. 2010; Shen et al. 2023; Boutsioukis, Verhaagen, et al. 2010). However, favourable fluid dynamic outputs should be interpreted as indicators of delivery rather than direct evidence of biofilm disruption, tissue dissolution or clinical safety. Their translational value depends on agreement with complementary chemical, microbiological and ex vivo evidence obtained in anatomically relevant models.

### 3.5 In Silico Endodontics Is No Longer Hypothetical

In silico approaches are no longer limited to theoretical discussion and are already being applied in endodontic research. Recent studies have used toxicity prediction, molecular docking, finite element models and structure‐assisted drug design to explore endodontic materials, microbial targets and inflammatory pathways before advanced biological validation (Malta et al. 2024; Lahoud et al. 2024; Geervani et al. 2025; Boreak and Bhandi 2022). This shift is important because it shows that computational methods are already contributing to real‐world practice. For example, Malta et al. (Malta et al. 2024) computationally compared compounds from hydraulic calcium silicate‐based sealers and AH Plus, reporting higher predicted toxicity and stronger interactions with inflammatory mediators for compounds related to AH Plus. Lahoud et al. (Lahoud et al. 2024) also identified finite element models as a relevant platform for in silico dental biomechanics, particularly in relation to personalised treatment planning, material selection and computer‐aided design and manufacturing. These studies illustrate how computational models may assist early material screening and biomechanical comparison before experimental translation.

This movement is also evident in the identification of therapeutic targets. A systematic review on *Enterococcus faecalis* proteins in endodontic infections showed that in silico methods have been used to screen ligands against targets such as Sortase A, MurA, cyclic di‐AMP synthetase and quorum‐sensing regulators, although methodological variability and the need for further in vivo validation remain important limitations (Geervani et al. 2025). Similarly, Boreak and Bhandi (Boreak and Bhandi 2022) used docking, molecular dynamics and Molecular Mechanics Poisson–Boltzmann Surface Area (MMPBSA) analyses to identify a potential IL‐8 inhibitor for endodontic pulpitis, illustrating how computational methods may generate therapeutic hypotheses that require subsequent in vitro, in vivo and clinical confirmation. Across these applications, the interpretation of computational outputs should remain aligned with the question each model was designed to address. Toxicity prediction may inform material screening, finite element analysis may support biomechanical comparison and molecular modelling may prioritise therapeutic candidates, but none independently establishes clinical effectiveness. Their contribution lies in reducing uncertainty before biological validation and directing subsequent research toward more plausible materials, designs, targets and protocols.

## 4 Conclusion

In silico research should be positioned in endodontics as a translational decision layer that supports laboratory, biological and clinical research. Its value lies in helping researchers move from broad virtual exploration to more focused physical validation rather than in generating large numbers of simulations. When used with appropriate methodological rigour, computational approaches may improve how endodontic questions are prioritised and how experimental resources are directed. The practical implications may be summarised as follows:

- In silico research can complement conventional models by exploring plausible variability before physical testing.
- The relevance of a virtual cohort depends on the quality, diversity and uncertainty of the data used to generate it.
- Computational outputs are most useful for prioritising targets, materials, designs, protocols and experimental conditions for subsequent investigation.
- Credible translation requires a defined context of use, transparent assumptions, verification, validation, uncertainty assessment and selective experimental confirmation.

## Author Contributions

José Evando da Silva‐Filho: Conceptualization; Data curation; Formal analysis; Investigation; writing – original draft; writing – review and editing. Eduardo Diogo Gurgel‐Filho: Conceptualization; Data curation; writing – original draft; writing – review and editing.

## Funding

The authors have nothing to report.

## Disclosure

AI usage statement: ChatGPT (GPT‐5) was used exclusively for grammar and spelling correction of text originally written by the authors. No content, analysis, interpretation or conclusions were generated by AI.

## Ethics Statement

The authors have nothing to report.

## Conflicts of Interest

The authors declare no conflicts of interest.

## Data Availability

Data sharing not applicable to this article as no datasets were generated or analysed during the current study.
